# Stem Cell-Based Acellular Therapy: Insight into Biogenesis, Bioengineering and Therapeutic Applications of Exosomes

**DOI:** 10.3390/biom14070792

**Published:** 2024-07-03

**Authors:** Mahmood S. Choudhery, Taqdees Arif, Ruhma Mahmood, David T. Harris

**Affiliations:** 1 Department of Human Genetics & Molecular Biology, University of Health Sciences, Lahore 54600, Pakistan; ms20031@yahoo.com (M.S.C.); taqdeesarif01@gmail.com (T.A.); 2 Allama Iqbal Medical College, Jinnah Hospital, Lahore 54700, Pakistan; ruhma_mahmood@yahoo.com; 3 Department of Immunobiology, College of Medicine, University of Arizona Health Sciences Biorepository, University of Arizona, Tucson, AZ 85721, USA

**Keywords:** exosomes, cellular therapy, acellular therapy, miRNA, biogenesis, bioengineering

## Abstract

The vast regenerative potential of stem cells has laid the foundation for stem cell-based therapies. However, certain challenges limit the application of cell-based therapies. The therapeutic use of cell-free therapy can avoid limitations associated with cell-based therapies. Acellular stem cell-based therapies rely on the use of biological factors released by stem cells, including growth factors and extracellular vesicles such as exosomes. Due to their comparable regenerative potential, acellular therapies may provide a feasible and scalable alternative to stem cell-based therapies. Exosomes are small vesicles secreted by various types of cells, including stem cells. Exosomes contain parent cell-derived nucleic acids, proteins, lipids, and other bioactive molecules. They play an important role in intra-cellular communication and influence the biological characteristics of cells. Exosomes inherit the properties of their parent cells; therefore, stem cell-derived exosomes are of particular interest for applications of regenerative medicine. In comparison to stem cell-based therapy, exosome therapy offers several benefits, such as easy transport and storage, no risk of immunological rejection, and few ethical dilemmas. Unlike stem cells, exosomes can be lyophilized and stored off-the-shelf, making acellular therapies standardized and more accessible while reducing overall treatment costs. Exosome-based acellular treatments are therefore readily available for applications in patients at the time of care. The current review discusses the use of exosomes as an acellular therapy. The review explores the molecular mechanism of exosome biogenesis, various methods for exosome isolation, and characterization. In addition, the latest advancements in bioengineering techniques to enhance exosome potential for acellular therapies have been discussed. The challenges in the use of exosomes as well as their diverse applications for the diagnosis and treatment of diseases have been reviewed in detail.

## 1. Background

Stem cells possess unique self-renewal, differentiation, and proliferative properties [1]. These inherent properties of stem cells make them ideal candidates for the regeneration of tissues and organs in the body [2]. In recent years, stem cells have been applied to treat many incurable diseases, such as cardiovascular, neurological, skin disorders, etc. Stem cells apply various mechanisms to regenerate tissues and organs of the body, such as trans-differentiation, control of apoptosis and inflammation, recruitment of other cells and tissues, angiogenesis, and secretion of several bioactive molecules [3]. Stem cell treatments have been broadly categorized into stem cell-based therapy and stem cell-based acellular therapy. Cellular therapy (cell-based therapy) aims to utilize cells, such as stem cells, to repair or replace damaged tissues or organs. However, limited proliferation, infusion toxicity, immunogenicity, oncologic complications, senescence, infection risks, and therapeutic delivery make cellular therapies questionable and limit their clinical use. Acellular therapy (cell-free therapy) has the potential to overcome these challenges and seems like a more feasible and efficient alternative to cellular therapy [4].

Acellular therapies aim to treat diseases by employing non-cellular biological factors that are released by the cells, such as growth factors, cytokines, and particularly the extracellular vesicles [5]. Exosomes, a type of extracellular vesicles, are spherical, lipid membrane-bound vesicles released by cells through the endosomal pathway [6]. They are nanoscale, and their molecular cargo is derived from the parent cells and includes DNA, different types of RNA, including microRNAs, lipids, and proteins. The endosomal route is the most commonly described mechanism of exosome biogenesis, in which primary endosomes are produced by the invagination of the cell membrane, followed by the accumulation of parent-cell-derived bioactive molecules inside the endosomes. Early endosomes develop into late endosomes, or multivesicular bodies (MVBs). Intraluminal vesicles (ILVs) are formed by the inward budding of the endosome membrane, which eventually forms exosomes within MVBs. The fusion of MVBs with the plasma membrane discharges the exosomes into the extracellular space. Exosomes exhibit distinct responses to various physiological signals and are subject to strict regulation, enabling them to perform their functions according to specific physiological conditions [7]. Initially, exosomes were considered rubbish bins for the removal of cellular waste. However, recent findings have explored their role in intercellular communication to alter biological processes and deliver drugs. Exosomes possess the ability to modify the physiological condition of cells or tissues, and their identification in body fluids as biomarkers provides opportunities for multiparameter diagnostic tests. Exosomes are suitable for use in developing acellular-based treatments due to their small size, ability to efficiently exchange their cellular components, in vivo biocompatibility, targeted delivery, regenerative potential, off-the-shelf storage, long-term stability, lower toxicity, and immunomodulation properties [8].

Although exosomes are secreted by almost all types of cells in the body, stem cell-derived exosomes are of particular interest in regenerative medicine. As exosomes exhibit the contents and properties of their parent cells, the biological and functional properties of stem cells make exosomes ideal candidates for repair and regeneration. Further, stem cells can be derived from a variety of readily available tissues, including adipose tissue, bone marrow, dental pulp, umbilical cord tissue, cord blood, etc. Stem cells are easy to cultivate in large numbers for the isolation of exosomes. Besides adult stem cells, human embryonic stem cells (hESCs) and induced pluripotent stem cells (iPSCs) also release exosomes. These cells possess superior self-renewal, differentiation, and proliferation potential compared to adult stem cells. Although their use in regenerative medicine as cell-based therapy is restricted, exosomes derived from these sources may be an alternative to these cells [9].

Exosomes do not self-replicate like stem cells, which reduces concerns about the risk of tumor growth following transplantation of stem cells. Exosomes can also be stored at ambient temperatures or kept frozen for extended periods following lyophilization. Their small size additionally permits filtration-based sterilization. Exosome delivery to target sites is not restricted to intravenous or intramuscular delivery; rather, they can be delivered at the site of injury in many ways, including, for example, inhalation of lyophilized or nebulized lung stem cell-derived exosomes for lung disease treatment. This makes exosomes a viable choice for the therapeutic application of stem cell-based acellular therapies [10]. Interestingly, exosomes can also be engineered to modify their contents, providing a unique opportunity to improve their natural properties for targeted therapeutic applications. Exosome engineering aims to improve the efficacy of exosomes for drug delivery, gene therapy, and immunomodulation vehicles by modifying their composition and surface features. New advances in bimolecular engineering have made it possible to generate and isolate tailored exosomes that are more stable, selective, and bioavailable [11].

Exosomes provide a non-invasive alternative to traditional treatments requiring invasive procedures or surgery. The non-invasive routes of exosome therapy include the intravenous route, subcutaneous route, intramuscular route, and aerosol sprays. Exosomes enclose and protect therapeutic compounds (such as DNA, RNA, and proteins) from degradation, selectively target certain cell types, and regulate the immune system [12]. These properties make them a preferred approach for treating various diseases. Exosomes have the potential to transform disease management, with important implications for modern medicine [13]. In addition, stem cell-derived exosomes can be readily available in times of care or emergency. Off-the-shelf availability of exosomes makes acellular therapy less expensive and more standardized. Nevertheless, there are several challenges in translating the exosome-based therapies into clinical practice. These include, but are not limited to, the following: standardizing exosome isolation and purification methods; understanding the uptake strategies and mechanisms of interactions between the exosome and target cell; and safety issues regarding exosome administration in humans. Notwithstanding these challenges, the potential of exosomes for therapeutic purposes remains a strong driving force for research and innovation. The goal is to develop treatments that fully utilize the potential of stem cell technology [14].

Overall, acellular therapies have broadened the scope of the application of stem cells for tissue restoration. This review covers the scope of exosomes by evaluating their major characteristics, biogenesis, and composition. It further explores in detail the isolation methods, bioengineering approaches to improve exosome potential, and various applications of exosomes in disease diagnosis and treatment.

## 2. Exosomes: A Type of Extracellular Vesicles

The term extracellular vesicles (EVs) was first used in 1970 to describe calcifying globules in epiphyseal cartilage [15]. EVs are nano-sized, lipid-bound vesicles that may influence a wide range of biological processes in the cells. EVs are secreted by prokaryotic and eukaryotic cells and regulate intercellular communication. These nanoparticles deliver DNA, RNA, microRNAs, and proteins to recipient cells [15]. EVs are categorized into three groups based on their size: apoptotic bodies, microvesicles (MVs), and exosomes. Apoptotic bodies are secreted by apoptotic cells and are relatively large as compared to microvesicles and exosomes (>1000 nm in diameter). Although they contain harmful waste substances, they may have a significant role in homeostasis regulation and signal transduction. MVs are irregular-shaped extracellular vesicles that are 100–1000 nm in size and evolve through the budding of the plasma membrane [16]. Exosomes are a type of EVs, that are enclosed in a phospholipid bilayer and range in size from 40 nm to 160 nm [10].

Exosomes were initially described as small EVs and were believed to serve as rubbish bins for the removal of unnecessary waste from the cellular environment. Further studies, however; found that exosomes are involved in intracellular signaling due to their ability to transport metabolites, proteins, and various nucleic acids between the cells. Recent research has provided evidence in support of this theory by identifying certain proteins that promote mechanisms of cellular transportation associated with various exosomal RNA species and the interactions of regulatory proteins. Exosomes are contained in various body fluids, including amniotic fluid, breast milk, saliva, cervical–vaginal discharge, semen, and plasma. Exosomes are secreted by various kinds of cells, such as epithelial cells, uterus macrophages, ESCs, endometrial cells, stem cells, oviductal epithelium cancer cells, follicular cells, placenta-derived cells, and dendritic cells. Exosomes reflect the biological state of donor cells and can serve as potential biomarkers for the diagnosis of many diseases [17].

Applications of exosomes are expanding day by day due to recent extensive research in this field [18]. Exosomes perform several roles, including facilitating intercellular communication, transmitting genetic information, regulating biological activities, and modulating immune responses. The diverse functions of exosomes also make them valuable for several therapeutic applications. Exosomes play a vital role in tissue regeneration by transporting cellular components and mimicking the physiological condition of donor cells in recipient cells. Furthermore, exosome contents are easily degradable or rendered inactive through a variety of pathways and sites. Target cells can safely contribute to immune control, tissue repair, tumor identification, and treatment, among other regulatory processes [19]. Exosomes have important roles in the treatment of a wide range of diseases, such as infections, cancer, and neurodegenerative diseases like Parkinson’s disease, Alzheimer’s disease (AD), lysosomal storage disorders, multiple sclerosis (MS), and prion disease. While applications of exosomes for diagnosing various diseases have been developed based on their role in disease pathogenesis, applications for treating cancer and autoimmune disorders are still being developed by leveraging their immune regulatory properties [20,21,22,23]. Because of their involvement in a wide range of physiological and pathological functions, EVs are not only potential disease indicators but also promising prospects for the development and implementation of new acellular (cell-free) therapeutics.

## 3. Exosomes Composition

**Figure 1:**
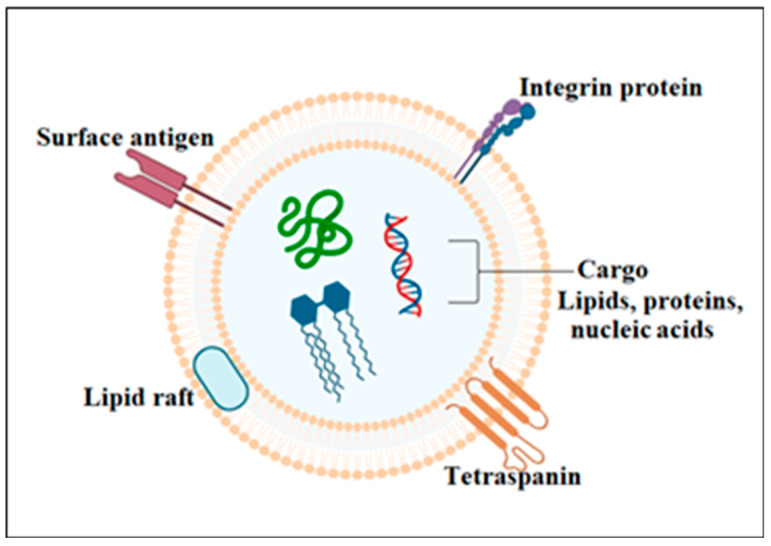
Structural organization of the exosome. Exosomal cargo includes nucleic acids, surface antigens, tetraspanin, integrin proteins, and lipid rafts. Nucleic acids (DNA, miRNA, mRNA, etc.) influence recipient cell gene expression and function. Surface antigens target cells and recognize immune cells. Tetraspanins (CD9, CD63, CD81, and CD82) facilitate exosome biogenesis and fusion with recipient cells. Integrin is a transmembrane receptor protein that acts as a cell adhesion molecule and controls exosome signaling and uptake. Lipid rafts of cholesterol and sphingolipids affect exosome stability and cargo sorting.

The cargo of exosomes encompasses surface antigens, signaling molecules, a wide variety of proteins, lipids, nucleic acids, and other signaling molecules such as cytokines (Figure 1). Various antigens are expressed on the exosome surface. These antigens bind with receptors on the recipient cells to start an intra-cellular signaling cascade. The glycolipids, glycoproteins, and cytokines carried by exosomes play an important role in intra-cellular signaling. Exosomal protein components fall into two main categories: public components and specific components. Public proteins are found in exosomes regardless of the type of cell from which they originate. These proteins are involved in the processes of vesicle formation, secretion, signaling, exosome uptake, and other general functions. They include membrane transport proteins, heat shock proteins (such as HSP70 and HSP90), fusion-related proteins (such as Rab and GTPases), transmembrane receptor proteins (such as integrin), members of the tetraspanin family (such as CD9, CD63, CD81, and CD82), and proteins related to the endosomal sorting complex required for transport (ESCRT) complex (such as Tsg101 and Alix) [18]. Specific protein components of exosomes are donor cell-specific and therefore represent the physiological and pathological conditions of the exosome-secreting cells. The exosomes released by antigen-presenting cells such as B cells, macrophages, and dendritic cells specifically contain MHC class 1 and MHC class II molecules. These proteins are important for immune regulation. Similarly, tumor-specific antigens (such as HER2 or PSA) are exhibited by the exosomes that are released by the cancer cells. The specific components of exosomes are identified by the types of parent cells and their surrounding environment. For example, exosomes released by lung cancer cells show an elevated level of epidermal growth factor receptor (EGFR) [24]. Plasma exosomes include x-box-binding protein 1 (NFX1) and cGMP-dependent protein kinase 1 (PKG1) [17].

The phospholipid bilayer membrane of exosomes comprises lipid components, including lipid rafts of cholesterol and sphingolipids. These structures have a role in exosome stability, exosome sorting, secretion, and communication with the host cell. The nucleic acid component of exosomes includes non-coding RNA, mRNA, ribosomal RNA, DNA, miRNA, transfer RNA, and short nuclear RNA. The nucleic acids in exosomes have important roles in understanding intra-cellular communication, gene regulation, disease diagnosis, regulation of innate and adaptive immune responses, and disease development and progression. The amount of nucleic acid within exosomes plays an important role in disease progression by transmitting specific genetic information. Exosomal RNA differs from RNA in the parent cell in several ways: (1) certain RNA species, such as miRNAs, are more abundant in exosomes than in the parent cell; (2) exosomal RNA is more stable due to the protective environment within exosomes; (3) parent cells have similar RNA profiles among different cell types, whereas exosomes have unique RNA profiles; (4) exosomal RNA regulates gene expression in recipient cells; and (5) exosomes selectively package and deliver specific RNA molecules for therapeutic purposes [25]. MicroRNAs are among the most prevalent RNA species in exosomes, and they have diverse functions, including exosome-mediated cellular communication, apoptosis, cell proliferation, cell migration, role in inflammatory reactions, and potential biomarkers in the diagnosis of certain diseases such as cardiovascular disease and cancer [26]. Many exosomal RNAs are associated with RNA-binding proteins to form ribonucleoprotein (RNP) complexes, such as Cas9 ribonucleoprotein. These RNP–protein complexes have diverse roles in several cellular processes, such as RNA translation, maturation, transport, and metabolism [25].

## 4. Biogenesis of Exosomes

**Figure 2:**
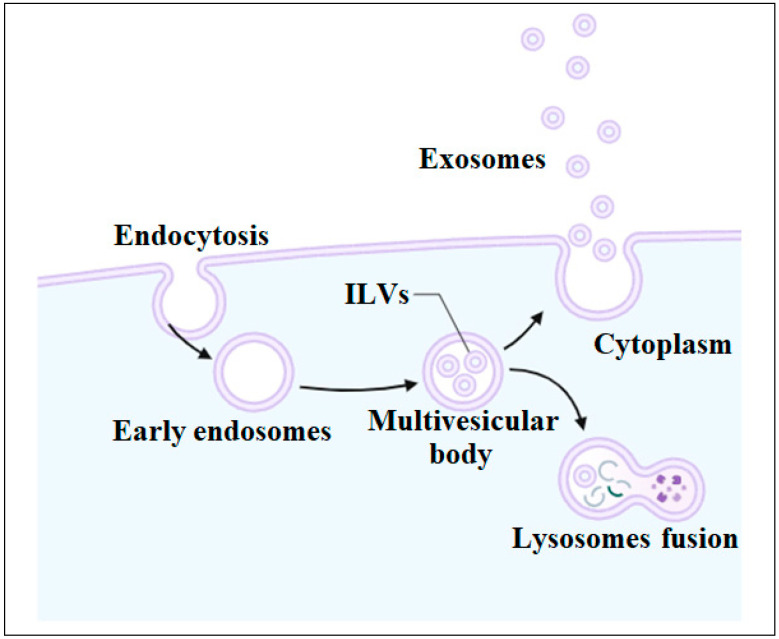
Biogenesis of exosomes. Exosome formation starts with endocytosis and the formation of early endosomes. ILVs are formed inside the MVBs. MVBs then fuse with the cell membrane and release exosomes into the extracellular matrix. Alternatively, MVBs may fuse with lysosomes and become a part of the degradation pathway.

Exosome biogenesis is a sophisticated process (Figure 2). It starts with the invagination of the cell membrane and the formation of early endosomes. These early endosomes mature into late endosomes called multivesicular bodies (MVBs) containing intraluminal vesicles (ILVs). The ILVs are released as exosomes by fusing with the plasma membrane and undergoing exocytosis. These exosomes have a diameter of around 40 to 160 nm. Protein, nucleic acids, and lipids are incorporated into exosomes during ILV formation. The endocytosis-sorting complex regulates the process of ILV formation. MVBs either fuse with the cell membrane and release exosomes into the extracellular environment or fuse with lysosomes and become a part of the degradation pathway. To date, several mechanisms involved in exosome biogenesis have been discovered [7,27]. The exact mechanism of the biogenesis of exosomes is not fully understood. However, some studies have suggested the involvement of syntenin and syndecan heparan sulphate proteoglycans in exosome formation. Syntenin is a cytosolic protein that is associated with the formation and release of exosomes. The transportation of exosomes entails an intricate interaction among several regulatory proteins, including syntenin, which communicates with its binding partners, such as Rab GTPases. Syntenin binds to Rab GTPases and regulates the fusion of MVBs with the plasma membrane, therefore promoting the secretion of exosomes into the extracellular space. Subsequently, the exosomes are transported to destination cells through the regulation of Ca^+2^ channels, ESCRTs, and cellular pH levels. Scientists have suggested that syntenin has the ability to transport and package miRNAs into exosomes, which in turn control intercellular communication by transferring miRNAs-carrying exosomes [28,29]. Syntenin was initially discovered as a binding partner of the C-terminal cytoplasmic domain of syndecan, thus called syndecan binding protein (SDCBP). Syntenin-1 has been shown to interact with syndecan, a heparan sulfate proteoglycan that regulates the function of different cell adhesion molecules and growth factors. Syndecan is also involved in the process of recycling endosomes and the formation of exosomes. During the synthesis of exosomes, syndecan heparan sulphate proteoglycans interact with the endosomal sorting complex and recruit syntenin, which helps facilitate membrane invagination, select the cargo of exosomes, and regulate MVB formation [30].

## 5. Classification of Exosomes

**Figure 3:**
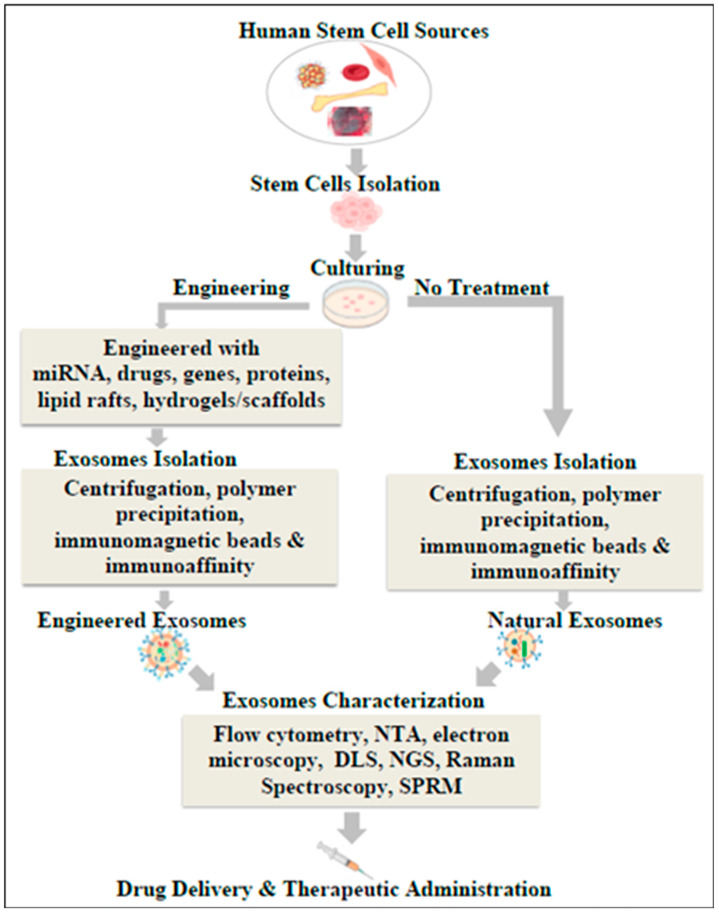
The figure depicts the isolation and modification of exosomes for drug delivery. Stem cells are isolated from readily available sources and cultured in a laboratory. The cultured cells on the right side of the figure are unmodified. Modifications to cultured cells using miRNA, drugs, genes, proteins, lipid rafts, etc. are shown on the left side of the figure. Natural and engineered exosomes are isolated using standard exosome isolation techniques. The isolated exosomes are subsequently characterized using flow cytometry, nanoparticle tracking analysis, and monochromatic laser beams. Characterized cells are then used for drug delivery and therapeutic administration.

Exosomes are classified into natural exosomes, engineered exosomes, and synthetic exosomes based on whether their contents are natural or have been modified using engineering and biotechnology approaches. Figure 3 shows a general overview of the isolation of natural exosomes and the production of modified exosomes for drug delivery.

### 5.1. Natural Exosomes

Natural exosomes are present in plant and animal cells, where they play roles in nutrient balance and intercellular communication. Natural exosomes can be derived from various sources, including body fluids, tumor cells, immune cells, and stem cells. Tumor exosomes possess immunosuppressive characteristics. They have a significant impact on the process of tumor growth and metastasis because they facilitate cell–cell contact. Since these vesicles are involved in cellular communication, they influence the growth and spread of tumors. Tumor-derived exosomes stimulate the development of CD4+ T cells into CD39^+^ regulatory T cells, which in turn leads to immunosuppression inside the tumor microenvironment (TME) [31]. Natural exosomes are also released by the immune cells. Dendritic cells (DCs), macrophages, and monocytes are the primary cell types that release exosomes naturally. Exosomes released by these immune cells are useful in the development of adaptive immunity. In addition, these exosomes can be employed in both therapeutic research and cancer diagnosis because they contribute to the advancement of diseases [32].

Stem cell-derived exosomes have a great interest in research and therapeutics due to their remarkable regenerative potential. The source of stem cells, however, must be chosen based on the treatment of a specific disease. This selection of exosome source influences not only the lipids and proteins present on the exosome surface but also their inside contents [33]. Stem cell-derived exosomes influence the self-renewal, immunomodulation, proliferation, and repair properties of the cells. Simultaneously, these exosomes hold considerable promise for cell-free, regenerative medicine. Interestingly, exosomes produced by ESCs promote signaling between stem cells and thus speed up tissue repair by enhancing cell proliferation. MSCs derived from different sources, such as bone marrow, adipose tissue, umbilical cord tissue, and muscles, have been used to isolate exosomes. Currently, MSC-derived exosomes have been extensively studied. They have been employed in the treatment of immunological disorders due to their immunomodulatory and regenerative properties [32].

### 5.2. Engineered Exosomes

Exosomes that are naturally formed can be altered for certain therapeutic purposes. The modifications of exosomes include the addition of drugs and other therapeutic substances, altering the surface charge to facilitate the immediate absorption of drugs, and loading the exosomes with specific contents. Researchers have previously altered exosomes derived from several natural sources, including mammalian biological fluids, to explore their potential use in biomedical applications. Exosomes can be modified by two methods, including interior modification, which involves changing the internal contents of exosomes, and surface modification, which alters the structure of the external surface [34].

#### 5.2.1. Modifications of Internal Contents

Drugs can be incorporated into naturally occurring exosomes using various internal modification techniques, such as electroporation, gene editing, sonication, and direct incorporation. The desired drug is introduced to the parental cells by incubation with the desired drug, which results in the encapsulation of the drug within the exosomes. The exosome-carried drugs are subsequently released into the desired population of cells in the body. However, it is difficult to control the loading efficiency with this procedure [35]. Gene editing techniques such as CRISPR-Cas9 can modify the parent cells [36], allowing the incorporation of therapeutic cargo such as proteins and RNA that cannot be directly integrated into exosomes. Such modification techniques can make their efficiency higher by minimizing drug loss, improving stability, Using a lipid extruder, an exosome and cargo mixture are extruded across a membrane with pore sizes ranging from 100 to 400 nm and loading capacity [29,37].

Different techniques are also used for the active incorporation of therapeutic drugs into exosomes. Electroporation, for example, is one of the active incorporation techniques where cargo enters inside exosomes through pores that are temporarily created in the phospholipid bilayer of the membrane. Using a lipid extruder, an exosome and cargo mixture is extruded across a membrane. A ruptured membrane allows the cargo to enter the exosomes. These techniques facilitate the diffusion of cargo into the core exosomes. The integrity of the membrane is restored following the diffusion of cargo [38].

#### 5.2.2. Surface Modifications

The surface of exosomes is crucial for their biodistribution, cell-specific targeting, and possible therapeutic applications. The required properties of exosomes can be induced by altering their surface, which improves cell targeting. The exosome surface can be altered by directly changing isolated exosomes or by directly modifying the parental cells that produce exosomes. Genetic engineering of parental cells can be used to modify the exosome membrane. Viral vectors genetically alter cells (cell membranes) by introducing the coding sequence of target ligands. Peptides are expressed on the membranes of exosomes. The peptide-containing exosomes are subsequently secreted by these cells [38].

### 5.3. Synthetic Exosomes

It is difficult to ensure uniform extraction and purification of natural exosomes with the required clinical grade for prospective use in therapeutic applications. It is necessary to apply acceptable modification strategies for the administration of genes, drugs, and other therapeutic substances, for which current methods may not be entirely adequate. Therefore, methods for creating synthetic exosomes to imitate natural exosome characteristics have been developed through biotechnology. The lipid membrane bilayer generation method and cell-based technique have been currently devised for the creation of synthetic exosomes [34].

The lipid membrane bilayer formation method works on the principle of a “bottom-up” approach, which is used to manufacture large and complex structures from small and simple components [39]. On the other hand, cell-based methodology works on the principle of “top-down” approach that is employed to produce smaller components from large and complex structures. Exosomes can be synthesized by two methods using this approach. One method pressurizes the living cells over hydrophilic microchannels of microfluidic devices. The living cells are broken down into smaller components in these channels and subsequently transformed into vesicles [39]. The second method produces vesicles of the same size by extruding cells over polycarbonate membrane filters with smaller pores [40].

## 6. Isolation of Exosomes

Exosomes exhibit diverse structures with a range of sizes, contents, and functions. The isolation of pure exosomes while maintaining their structure is challenging. Different techniques, such as differential centrifugation, polymer precipitation, commercially available exosome isolation kits, sequential filtration, the use of immunomagnetic beads, and immunoaffinity, have been devised to effectively isolate exosomes from various sources [29]. Table 1 shows advantages and disadvantages of various exosome isolation techniques.

### 6.1. Differential Ultracentrifugation

The first method used for exosome isolation was differential ultracentrifugation, which continues to be the most reliable and widely accepted approach. Differential ultracentrifugation is a cost-effective method that utilizes a single ultracentrifuge and requires minimal or no sample preparation. It also requires little technical expertise. Like other centrifugation procedures, the isolation of exosomes relies on their density, size, and shape. Larger and denser particles settle out first. Initially, stem cell culture medium is centrifuged at low speed (500× *g*) to isolate cells, large particles, macromolecular proteins, and cellular debris from the culture fluid. The supernatant is then separated and centrifuged for 70 min at 100,000× *g*. Increasing the duration of centrifugation during the 100,000× *g* spins can enhance the exosomal yield. However, centrifuge durations of more than 4 h may cause mechanical damage to exosome membranes, resulting in sample contamination with released proteins. Differential ultracentrifugation is a time-consuming procedure that necessitates the use of large quantities of samples. This poses a difficulty in effectively managing several biological samples within a restricted timeframe [41,42].

### 6.2. Sequential Filtration

It involves using a sequence of filtration processes to enrich exosomes. The procedure starts with the use of a 100 nm filter to remove large particles, cells, and cellular debris. Particles of diameter larger than 100 nm, such as exosomes, easily penetrate the filter as long as they are flexible. However, rigid components are eliminated. due to their flexible structure. The components of cellular debris are removed by filtration. The filtrate is subjected to tangential flow filtration using a 500 kDa MWCO membrane in order to purify the exosomes, eliminate soluble proteins, and remove other small molecules and impurities. The remaining solution containing the exosomes is then sterilized with a 100 nm track-etch filter to remove any impurities from bacteria and other microorganisms. This method has several benefits, such as the fact that the process is streamlined and automated, it can efficiently isolate exosomes from large volumes of media, and the resulting exosomal product can be preserved while retaining its biological activity. Nevertheless, the procedure is time-consuming due to the need to change filter papers, which poses challenges in standardizing the isolation process. In addition, small exosomes have the ability to traverse filters, leading to the loss of valuable material [43].

### 6.3. Polymer Precipitation

Polymer precipitation is a preferable method for the isolation and separation of exosomes. Polyethylene glycol (PEG) medium is commonly used in this method. PEG is a water-repellent polymer that binds to water molecules, resulting in the precipitation of exosomes. The exosome vesicles that have been formed after precipitation can be collected with centrifugation and subsequently used for various types of analysis. The method has the advantage of processing large volumes of samples (ranging from 100 microliters to several milliliters) quickly with an easy-to-use approach. The strategy to isolate exosomes is simple, efficient, and does not require costly equipment or technical knowledge. The primary limitation of this method is a lack of sensitivity, which prevents it from being used in clinical applications. PEG polymers not only precipitate exosomes but also other extracellular vesicles. Thus, preliminary steps like centrifugation or filtration are necessary to minimize contamination in the final product of exosomes [42].

### 6.4. Exosome Isolation Kit

Several exosome isolation kits have been developed to obtain a pure population of exosomes. For example, the ExoMir Kit, manufactured by Bioo Scientific in Austin, TX, USA, is a commercially available exosome isolation kit designed to isolate exosomes depending on their size. A syringe is used to hold two membranes, one measuring 200 nm and the other measuring 20 nm. The 200 nm filter is positioned at the top, while the 20 nm filter is put at the bottom. Typically, the sample undergoes low-speed centrifugation to separate cellular debris, cells. Proteinase K was then added to facilitate the degradation of larger particles and avoid membrane blockage. The sample is filtered in the mentioned syringe following the initial centrifugation step. Vesicles larger than 200 nm are retained above the first filter, while vesicles between 200 nm and 20 nm are trapped between the two filters in the syringe. Vesicles smaller than 20 nm flow through the syringe and are discarded [44]. The method has several advantages, such as a fast isolation procedure, being suitable for small volumes, and the high yield and purity of isolated exosomes. However, isolation of large-sized exosomes is difficult with this method. It also has a high cost and may not be suitable for downstream applications [45]. Various other commercial kits, such as ExoQuick by System Biosciences (Palo Alto, CA, USA) and Total Exosome Isolation Kit by Thermo Fisher Scientific (Waltham, MA, USA), have been developed and are now available for isolating exosomes.

### 6.5. Immunomagnetic Beads

A type of spherical particle coated with monoclonal antibodies is known as an immunomagnetic bead. These beads can be used to separate exosomes on the basis of the interaction between coated antibodies on the bead and receptors on the surface of the exosomes, such as CD9, CD63, and CD81. The immunomagnetic beads were initially coated with antibodies that bound to the surface of the exosome through receptors. The exosomes are isolated from the sample by inducing targeted migration of these complexes under the influence of a magnetic field. This method has many benefits, including low primary sample quantity, no chemical contamination, and high accuracy. However, the method encounters a lack of recognition of specific antibodies, and hence targeted exosomes may not be obtained. Consequently, this limitation restricts its use in clinical settings [42,46].

### 6.6. Affinity Chromatography

The foundation of affinity chromatography is based on the ability to use chemical or physical properties to separate molecules according to their biological activity. The fundamental idea behind this technique is that the interaction between the ligand and antibody separates the targeted molecule. The sample in the buffer solution is passed through the column after the ligand is covalently attached to the solid support known as the matrix. In the meantime, the interaction between targeted antibodies and the ligands occurs in the solid support. Subsequently, the desired molecule is removed from the column while the target molecule remains there [29]. Compared to other isolation methods, this method has the advantage of isolating exosomes from specific sources and other EVs. However, this method also has limitations, including the requirement for prior filtration or centrifugation due to the complexity of biological fluids, the inability of antibodies to recognize antigen within enclosed exosomes, the dependence of method specificity on the specificity of the used antibody, and a lower yield of isolated exosomes [43].

## 7. Characterization of Exosomes

The goal of exosome characterization is to recognize exosomes and identify the contents that an exosome is transmitting to neighboring cells. Exosomes are characterized on the basis of their morphology, physical properties, and contents. Electron microscopy, flow cytometry, nanoparticle tracking analysis, and dynamic light scattering methods are commonly employed for morphology assessment and other physical characterization of exosomes. Other methods, such as qPCR, next-generation sequencing, Raman spectroscopy, and surface plasmon resonance microscopy (SPRM), have been employed to analyze the exosomal contents.

### 7.1. Electron Microscopy

An electron microscope uses a beam of accelerated electrons to magnify small objects and investigate the morphology of biological specimens. Transmission electron microscopy (TEM) and scanning electron microscopy (SEM) are commonly used for the assessment of exosome morphology. The distinction between these methods depends on the specific electrons that are detected. In TEM, the electrons that traverse the sample are detected, whereas in SEM, the scattered electrons are detected. The electron beam then generates high-resolution images that are less than one micron in size. The particles in the sample generate dark areas, known as shadows, on the fluorescent screen, thus generating an image. More specifically, the electrons that are dispersed in various directions are captured and measured for image formation. Exosomal vesicles seen in TEM and SEM often have a central divot. This is probably a result of the dehydration process involved in the sample preparation necessary for TEM and SEM. Regarding exosomes, both TEM and SEM show an identical distribution of particle sizes, though they exhibit slightly different morphologies [47].

### 7.2. Flow Cytometry

Flow cytometry is most frequently employed to study the chemical and physical characteristics of exosomes. Once bound to the surface of beads, the exosomes are subjected to fluorescently labeled antibodies that specifically target the CD9, CD81, or CD63 antigens, which are known to be present on the surface of exosomes. Subsequently, stained exosomes cross the laser of the flow cytometer, which generates a fluorescent signal that is captured by the relevant detector. This method enables both efficient analysis of exosomes and the ability to quantify or classify exosomes depending on specific antigen expression. Flow cytometry uses samples at low concentrations. It works efficiently and produces quick results [29].

### 7.3. Nanoparticle Tracking Analysis

Nanoparticle tracking analysis (NTA) is often used to identify the size and concentration of exosomes. NTA uses routes of exosome motion to measure particle velocities based on the optical particle tracking technique. The laser light undergoes scattering when it interacts with the particles in the chamber. The scattered light is then captured by a microscope equipped with a camera. The camera is positioned above the microscope and records the motion of particles, which is then analyzed by the NTA software v2.3 to identify the size and concentration. This method can accurately measure the size of particles ranging from 10 to 1000 nm in diameter. An additional advantage is that, once the measurements are finished, the particles can be readily obtained in their original form. However, this method requires the optimization of analytical parameters and the use of sample volumes of around 0.5 mL [43].

### 7.4. Dynamic Light Scattering

Dynamic light scattering (DLS) measures the particles in a solution with sizes ranging from 1 nm to 6 µm. Monochromatic, coherent laser beams are used to scan the sample to evaluate the exosome size. DLS predicts particle size and concentration by analyzing scattered light from exosome motion, similar to NTA. However, DLS predicts particle size by evaluating changes in the intensity of scattered light rather than depending on the diffusion of particles. Monodisperse systems, or suspensions of individual particles, yield measurements that are more precise. When large molecules are present in the solution, even at low concentrations, a number of issues arise in the diagnosis of microscopic particles since the solution is unsuitable for complicated measurements [29].

### 7.5. Next Generation Sequencing

Exosomes are analyzed for their genetic composition using next-generation sequencing (NGS) and qPCR. NGS was implemented in 2013 to study exosomal RNA. The transcriptomic analysis of human breast cancer cell exosomes using NGS revealed that the majority of the RNA discovered in these exosomes consists of fragments of 28S and 18S ribosomal RNA (rRNA). Simultaneously, additional studies were performed on the short RNA transcriptomes of exosomes in human saliva. The data indicate that exosomal RNA comprises not only messenger RNA (mRNA) and microRNA (miRNA), but also small RNA (sRNA) and piwi-interacting RNA (piRNA). Furthermore, as exosomes include several miRNAs, it is important to assess their abundance in exosomes using both qualitative and quantitative methods. Quantifying the levels of DNA and RNA in exosomes necessitates the use of high-throughput techniques, such as microarray and next-generation sequencing (NGS), due to the intricate genetic information of exosomes [48].

### 7.6. Surface Plasmon Resonance Microscopy (SPRM)

SPRM is an efficient method that can identify exosomal membrane proteins and give real-time information on the kinetics of protein binding. In recent years, standalone plasmonic sensors have frequently been used to detect exosomal membrane proteins. In this technique, the angle of incidence of light determines the amount of light that is reflected or absorbed when incident polarized light couples to plasmons in metal during measurement. The camera captures the reflected light and correlates it with the availability of membrane proteins. The concentration of suspended exosomes is measured by initially translating the SPR response into the mass that is bound to the surface. A formalism that describes diffusion-limited binding under controlled flow circumstances is then used to establish a relationship between the increase in mass absorption and the concentration of exosomes in solution over time [49].

### 7.7. Raman Spectroscopy

Raman spectroscopy can distinguish exosomes based on lipid composition and other surface modifications. Raman spectroscopy depends on the scattering of photons to analyze the vibrational and rotational characteristics of molecules. A high-intensity laser beams at the sample, causing the incident light to disperse as it is diverted by the sample. A minimal quantity of light disperses at wavelengths distinct from the wavelength of the laser source, known as Raman scatter. The scattering of light depends on the chemical composition of the substance being analyzed. The Raman spectrometer captures a spectrum that exhibits peaks of intensity that correspond to certain bond vibrations. Raman spectroscopy is a label-free method that exhibits a high level of selectivity. Through the utilization of this method, it is possible to differentiate the various lipid types found in exosomes as non-protein indicators. Laser tweezers Raman spectroscopy and surface-enhanced Raman spectroscopy are two setups needed for exosome lipid analysis using Raman spectroscopy [50].

## 8. Applications of Exosomes

Stem cell-derived exosomes have a special role in the regeneration of tissues and organs in the body as they inherit the therapeutic characteristics of their parent cells, i.e., stem cells, which have the potential to repair, replace, and regenerate damaged organs and tissues [2]. As stem cells exhibit anti-inflammation, immunomodulation, and tissue regeneration, exosomes derived from them may also exert the same effects. Being an important bioactive molecule for acellular therapy, exosomes have a considerable role in regenerative medicine applications including neurodegenerative diseases, cardiovascular diseases, orthopedic diseases, immune regulation and cancer treatment, and drug delivery systems. In addition, exosomes may act as biomarkers for the onset, progression, and diagnosis of diseases. Exosomes have also been employed for the delivery of drugs to target cells [51].

### 8.1. Regenerative Medicine

Regenerative medicine is a multidisciplinary field of medicine that focuses on the regeneration, replacement, and repair of damaged cells, tissues, or organs [2]. It includes the application of therapeutic cells such as stem cells, tissue engineering technologies, gene therapy, and biomaterials as scaffolds [2]. The therapeutic utility of cell-based therapies is limited due to the above-mentioned challenges. Considering these limitations, exosome- based therapy (acellular therapy) is preferred because of its fewer side effects and comparable benefits. In addition to the off-the-shelf availability of exosomes, low immunogenicity, and isolation from a variety of autologous and allogenic sources, health care providers have focused on the possible use of exosomes in regenerative medicine applications. Exosomes are useful in functional recovery and tissue regeneration in a variety of diseases, including lung problems, liver fibrosis, colitis, osteoarthritis, myocardial infarction, retinal diseases, and spinal cord injury. Exosomes originating from stem cells can be helpful in the regeneration of blood vessels, bones, muscles, cartilage, nerves, and dentin. Interestingly, exosomes can also be modified to exhibit desired regenerative properties [52].

### 8.2. Neurodegenerative Diseases

Neurodegenerative disorders cause slow loss of brain cells, resulting in a decline in motor, cognitive, and sensory abilities over time. Protein aggregation, altered protein regulation, cytoskeletal abnormalities, metabolic imbalance, genetic mistakes, inflammation, and neuronal death are all hallmarks of these diseases. Exosomes can successfully cross the blood–brain barrier, making them promising candidates for brain tissue regeneration and drug delivery agents to treat neurological disorders. Alzheimer’s and Parkinson’s diseases are the most common neurodegenerative diseases. Currently, only symptom-relieving drugs such as N-methyl-D-aspartate (NMDA) antagonists and cholinesterase inhibitors are available to treat Alzheimer’s disease. However, the blood–brain barrier and pharmacokinetic difficulties present significant challenges in the effective delivery of drugs to the brain. Exosomes, being small-sized vesicles that contain powerful biomolecules, may circumvent these constraints. Researchers have derived exosomes from MSCs and combined them with rabies virus glycoproteins that are specific to the central nervous system. These modified exosomes were administered to the brains of transgenic Alzheimer’s mice. The modified exosomes improved their learning and memory concomitant with decreased Aβ deposition and increased distribution to the cortex and hippocampus [53].

Independent studies have evaluated the potential benefits of exosomes in the treatment of neurological disorders. For example, studies using an animal model of middle cerebral artery blockage have demonstrated the role of MSCs in upregulating the expression of miR-133b (contained in the exosome) in the ipsilateral hemisphere. After being exposed to ipsilateral ischemia tissue extracts in vitro, this miRNA was expressed more in MSC-derived exosomes than MSCs. Primary cultured neurons and astrocytes that were exposed to the MSC-derived exosome-enriched materials have likewise shown increased expression of miR-133b [53]. The administration of normoxic MSC-derived exosomes has improved memory and cognition deficiencies, reduced brain Aβ levels, and reduced plaque deposition in Alzheimer disease. These outcomes are associated with increased IL-4 and IL-10 and decreased TNF-α and IL-1β [54]. Another study demonstrated that exosomes derived from BM-MSCs decreased the number of deteriorating axons in the optic nerve and showed neuroprotective advantages in chronic ocular hypertension-induced glaucoma [55].

### 8.3. Skin Disorders

Skin consists of sweat glands, hair, blood arteries, lymphatics, and nerve muscles and provides a protective barrier against external invaders. However, various internal factors, such as psychological factors, genetic factors, and other medical diseases, and external factors, such as, infection, sunlight, and invasion by irritants, collectively contribute to dermatological conditions and skin injuries. For many years, cell-based treatments, particularly MSC therapy, showed great potential for tissue regeneration. Recent research suggests that cells such as MSCs primarily repair tissue function via paracrine mechanisms, with exosomes playing a prominent role. Exosomes released by MSCs are considered safer and more stable compared to the actual MSCs themselves when it comes to the handling of live cells. In severe burns and diabetes-induced wounds, the skin’s ability to properly recover is compromised, which may result in skin ulcers or other infections. MSC-derived exosomes provide significant therapeutic potential in the regeneration of skin tissues due to their wide availability, ability to grow easily in a laboratory setting, and minimal risk of rejection after being transplanted into the body [56]. Exosomes derived from bone marrow mesenchymal stem cells (BM-MSC) can expedite wound healing by enhancing the formation of new blood vessels in dermal lacerations [57]. This reparative effect is attributed to the presence of diverse angiogenesis-related active factors within BM-MSC exosomes. In addition, adipose-derived mesenchymal stem cell (AT-MSC) exosomes enhance the growth and movement of certain cells in skin fibroblasts [58].

### 8.4. Cardiovascular Diseases

Cardiovascular disease (CVD) is the main cause of death worldwide. Acute myocardial infarction (AMI) and ischemic heart failure (IHF) are the most common cardiac diseases. Although bypass surgery or coronary interventions increase the overall survival rate of AMI patients, a significant number of these individuals have heart failure due to the loss of cardiomyocytes and associated tissue remodeling [59]. Exosomes produced from stem cells, specifically MSC-derived exosomes, have effectively treated patients with acute myocardial infraction and ischemic heart failure. Nevertheless, later studies reported that the majority of MSC-derived exosomes that were delivered intravenously were trapped in the lung rather than successfully engrafted in the heart [60]. It was discovered that the supernatant of induced MSCs generated from human embryonic stem cells (ESCs) included nano-sized particles, which were identified as exosomes. When given to a mouse model of cardiac ischemia injury, these exosomes significantly decreased the size of the damaged heart tissue. In addition, the researchers also introduced exosomes that were released by MSCs produced from human ESCs into a mouse model of acute myocardial infarction. They observed significant improvements in heart function [61]. Interestingly, exosomes derived from human cardiac progenitor cells (CPCs) have the potential to restore normal cardiac function. For example, clinical studies have introduced the exosomes obtained from human CPCs in a rat and mouse model of AMI that effectively reduced cell death, promoted the formation of new blood vessels, and enhanced heart function. Moreover, exosomes derived from CPCs have higher concentrations of some microRNAs (miRs), including miR-210, miR-132, and miR-146a-3p. The administration of these miRs partially imitated the positive effects of CPC-derived exosomes on heart function [62].

### 8.5. Diabetes Mellitus

Diabetes mellitus is a metabolic disorder that is largely defined by high blood sugar levels. It is mostly caused by abnormalities in the production of insulin, the hormone responsible for maintaining blood sugar levels. Prolonged and persistently high levels of glucose in the blood may lead to the impairment of body organs. Conventional use of insulin and oral medications is not effective in preventing the gradual loss of pancreatic beta cells in diabetes. These treatments simply alleviate the symptoms of the disease. MSC-Exos have been recommended as a new treatment approach for diabetic patients due to their greater efficacy compared to MSCs [63]. Research has also shown that exosomes produced from AT-MSCs have the ability to modulate the immune response of T cells in type 1 diabetes (T1DM) without affecting the growth of lymphocytes [64]. Bone marrow mesenchymal stem cell-derived exosomes (BMMSC-Exos) also have the potential to significantly decrease blood glucose levels and increase plasma insulin levels in T1DM [65].

Promoting the regeneration of islets and improving the production of insulin is essential for the recovery of type 2 diabetes mellitus (T2DM). UCBMSC-Exos derived from mesenchymal stem cells in umbilical cord blood were shown to promote the proliferation of Langerhans islet cells in mice with STZ-induced diabetes. As a result, the pancreas underwent regeneration and exhibited improved insulin production via regulating the Extl3-Reg-cyclinD1 pathway. Hyperglycemia in diabetic individuals promotes increased oxygen consumption in β-cells, which leads to hypoxia and a decrease in PDX1 and MAFA. This ultimately results in apoptosis and malfunction of β-cells [66]. Human umbilical cord mesenchymal stem cells (HucMSC-Exos) have the ability to reduce the destruction of β-cells and reverse insulin resistance in peripheral tissues. HucMSC-Exos have been shown to reduce the death of β-cells under low oxygen conditions by reducing the stress on the endoplasmic reticulum caused by low oxygen and suppressing the p38 MAPK signaling pathway in β-cells. This effect is facilitated by the high levels of miR-21 present in HucMSC-Exos [67].

Interestingly, MSC-Exos have also been used in the treatment of many complications associated with diabetes, including diabetic retinopathy, diabetic erectile dysfunction, diabetic osteoporosis, cognitive impairment, diabetic cardiomyopathy, and peripheral neuropathy [63]. HucMSCs, for example, ameliorate the degeneration of retinal neurons, inhibit microvascular disease, and decrease the loss of neuronal cells and damage to retinal blood vessels [68]. BMMSCs have been used to address cognitive impairment through the reduction in oxidative stress and the enhancement of synaptic density. Moreover, they impede the proliferation of microglia in the brain [69].

### 8.6. Immune Regulation and Cancer Treatment

The immunological response is one of the protective mechanisms of the body against disease causing substances and biological agents. Cancer immunotherapy continues to be a focus of the scientific community due to its capacity to increase immunity, adapt to various forms of cancer, and have a lasting effect. Exosome-based treatment is a potentially innovative method of cancer immunotherapy since it can be used to deliver anti-cancer drugs and trigger anti-cancer immune responses in target cells [70]. Exosomes, produced specifically from immune cells and tumor cells, have a distinct composition that is directly related to immunotherapy for cancer treatment. Exosomes have the ability to transport their cargos to specific cells, altering the phenotypic and immune-regulating capabilities of those cells. Exosomes play dual roles by contributing to the development of cancer as well as being therapeutic agents against cancer [71].

The deployment of exosome-based therapies for cancer treatment is growing as a potential area of research. Exosomes secreted by cancerous cells have the ability to modify several stromal cell types in order to stimulate autocrine VEGF signaling in endothelial cells, which in turn promotes tumor angiogenesis and encourages the proliferation and invasive nature of cancer cells [72]. Exosomes have the ability to express TGF-β and PD-L1 molecules that are involved in immunosuppression. On the other hand, exosomes formed by cancer cells have the ability to dampen the immune system by limiting CD8+ T cell activation and proliferation while boosting the formation of regulatory T cells [73]. Exosomes generated by tumors have been extensively researched in relation to their effect on a number of cancer types, including melanoma, renal, hematological, and breast cancers [74]. A study on pancreatic cancer discovered that exosome proteins with reduced miRNA levels might function as agonists to selectively activate cytokine-induced killer cells [75]. Research on non-small cell lung cancer (NSCLC) has shown that exosomes produced from tumor cells overexpressing Rab27a can promote DC maturation by upregulating MHC II and the CD80 and CD86 costimulatory markers. This effect increases the proliferation and responsiveness of CD4+ T cells both in vivo and in vitro [76]. Exosomes produced from leukemia cells that have been repressed by TGF-β1 improve the activity of DCs by secreting TNF-α and interleukin (IL)-12p70, as well as by upregulating MHC II and costimulatory molecules [77].

Exosomes are not only tailored to be antigen/drug carriers, but they also have the potential for the development of cancer vaccines. Exosome-based immunotherapy is now in its initial phase of clinical trials, and there are presently no global standards for monitoring the production and use of this innovative type of therapeutic agent. Prior to the use of exosomes in clinical applications, it is essential to establish quality classifications and criteria for biopharmaceuticals that ensure the safety of exosomal therapy [71].

### 8.7. Drug Delivery Systems

Exosomes can serve as drug carriers because of their ability to encapsulate and protect therapeutic substances. Treatment can be made more targeted and effective by engineering them to deliver drugs, small molecules, or RNA to particular target cells. Conventional treatments may have the potential to harm healthy cells during delivery. As therapeutic efficacy increases, drug delivery by exosomes becomes a more significant mechanism when compared to traditional free-form drug administration. Drug delivery vehicles need to fulfill several requirements in order to distribute drugs successfully. A sufficient quantity of the drug must be able to be integrated into drug carriers while also guaranteeing targeted drug administration and useful therapeutic outcomes. They must be extremely low in toxicity as well as biocompatible with the immune system’s response in order to avoid deterioration before reaching their targets [78]. These features are obvious when exosomes are used as drug delivery systems. They can also cause a low rate of persistent retention in organs and tissues and have very little or no toxicity. Additionally, they have the ability to infiltrate plasma membranes and a variety of biological fluids, allowing them to transport therapeutic substances into the cytoplasm of target cells. Exosomes therefore have great promise as therapeutic drug delivery systems, especially because they are able to pass through the blood–brain barrier [78].

A variety of biomaterials have been employed to protect, support, and improve the local administration of exosomes, with the aim of optimizing their therapeutic effectiveness. For example, exosomes have been integrated with tissue-engineered hydrogel/scaffold to improve their therapeutic potential and facilitate tissue regeneration and repair. Tissue engineered scaffolds and hydrogels provide both structural support and an optimal environment for cellular proliferation and tissue regeneration [79,80]. Combining exosomes with scaffolds enables the controlled and prolonged release of biomolecules. Engineered scaffolds ensure exosome delivery to the desired tissues. A biological scaffold containing exosomes needs to retain them at the implantation site while preserving their activity and ensuring constant release. The primary methods used for creating composites include physical adsorption, chemical cross-linking, 3D printing, freeze-drying, and specific binding. Various composite scaffolds exhibit suitable porosity, high biocompatibility, and the ability to constantly release exosomes in vivo [79,81].

Hydrogels are biomaterials composed of a 3D hydrophilic network of biodegradable polymers. They are widely used in regenerative medicine and tissue engineering due to their ability to facilitate cell adhesion and stimulate tissue regeneration. Moreover, they have the ability to imitate the mechanical and metabolic characteristics of the extracellular matrix (ECM), which serves as the inherent scaffold for cells in tissues. Tissue-engineered hydrogels encapsulate exosomes, creating a hydrated environment that promotes cell proliferation, protects them from degradation, and facilitates the controlled release of exosomes at specific target cells. Certain hydrogels, however require external gelation procedures in which heat and ultraviolet radiation are applied. These processes make them unsuitable for encapsulating small extracellular vesicles (sEVs), like exosomes. As these techniques can damage the RNAs present in sEVs, a specialized PEGylated poly (glycerol sebacate) acrylate (PEGS-A) injectable hydrogel cage has been developed to quickly immobilize within the body at normal temperature, to controllably release sEVs by surface erosion, and to undergo self-shaping in a water-based environment. The unique properties of sEVs enable the maintenance of their integrity and the preservation of sensitive mRNAs that were integrated before implantation. This, in turn, enhances the translational efficiency of the genetic material of SEVs. In addition, the use of PEGS-A with tailored modifications of pre-polymers and cross-linking agents enhances the prolonged retention of therapeutic exosomes in the damaged areas and improves their sustained activity to promote efficient regeneration [80].

**Figure 4:**
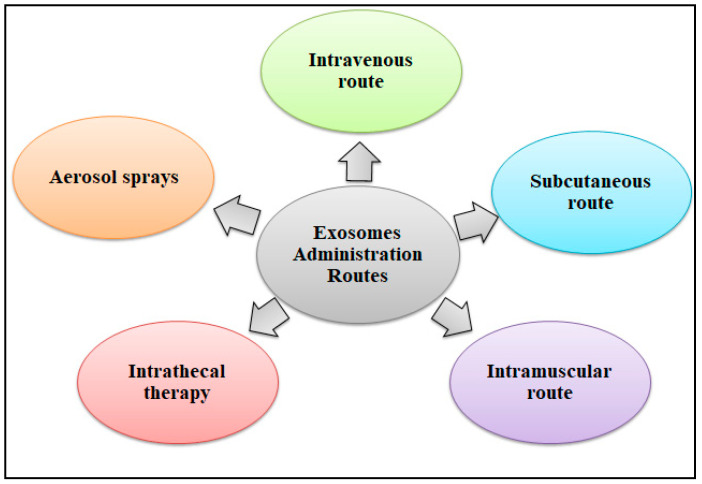
Methods for delivering exosomes to desired targets. Intravenous route: A needle containing therapeutic exosomes is inserted directly into a vein. Aerosol spray: inhalation of a tiny mist, droplets, or spray containing exosomes. Intrathecal administration: provide exosomes with an intrathecal injection, which is inserted into the spinal canal. Intramuscular route: a large volume of exosomes is injected into the muscle of the upper arm, thigh, or buttock. Subcutaneous route: drugs containing exosomes are injected just beneath the skin.

Exosomes can be used to develop safer, cell-free treatments than those that require cell therapy, which is another benefit. Therapeutic compounds must be successfully integrated into exosomes for efficient delivery. Various methods for delivering exosomes to their desired targets have been devised. The most often used route is the intravenous (IV) route, even though this approach has a quick clearance in the kidney and liver. Cardiovascular, neoplastic, and orthopedic diseases were among the disorders for which this approach was frequently employed. Subcutaneous (SC) injections are utilized for aesthetic and cosmetic purposes, whereas intramuscular (IM) injections have historically been used for neuromuscular and musculoskeletal disorders. The SC or IM method is chosen because of the convenient injection area and the dosing volume. When treating Parkinson’s disease, Alzheimer’s disease, Creutzfeldt-Jakob’s disease, and other neurological diseases, intrathecal therapy is the recommended approach. Wounds and ulcers have been treated using local aerosol sprays, which is also the suggested path for hair development and regeneration in age-related therapies [78,82]. The various exosome-based drug administration routes have been depicted in Figure 4.

### 8.8. Exosomes as Biomarkers for Diagnosis

The molecular profile and dynamic nature of exosomes have a significant role in disease pathology and can be employed as suitable indicators in the early diagnosis of disease. Exosomes derived from body fluids are laden with biomolecules that depict the features of parent cells. Therefore, isolated exosomes from body fluid can be used to develop distinctive diagnostic tests for the detection of diseases [83]. Exosomal proteins play a critical role in the diagnosis of numerous diseases, including cancer, cardiovascular diseases, and liver disease. Molecules like nucleic acid and protein within exosomes are highly significant for use as biomarkers. Cholesterol and sphingomyelin lipids, as well as proteins such as TSG101, CD63, CD9, and CD81, are examples of biomarkers. Although membrane scaffold proteins have been used for exosome characterization, their varying expression among different types of cancer, makes them promising biomarkers for cancer diagnosis [84]. Fettuin-A and proteins involved in the epidermal growth factor receptor (EGFR) pathway are used as indicators of acute renal disease [84]. Exosomes contain a large number of microRNAs, which are widely used as disease biomarkers. miR-1246 and miR-21, for example, are used for the diagnosis of breast cancer. Similarly, miR-21, 107, 181a-2, 301a, 141, 331-3p, 625, 326, and 2110 are used for the diagnosis of prostate cancer, and miR-638 is a biomarker of colorectal cancer cells. Heart failure and myocardial infarction patients have higher levels of miR-499, 133, 208, 192, 194, and 34a, which makes them diagnostic indicators for cardiovascular diseases [29].

Scientists have also isolated exosomes from different biopsies to study the development of diseases, assess the effectiveness of medical treatments, and explore improved therapeutic approaches. One such clinical study examined the urine exosomes of 24 individuals for the identification of hypertension. Ang II and other hormone angiotensin (Ang) peptides were measured in patient plasma to evaluate the renin–angiotensin–aldosterone system, which controls the abnormal renal sodium concentration that causes this medical condition. Urinary exosomes were selected as a primary outcome measure due to their role as markers of renal function, thus providing suitable guidelines to evaluate the therapeutic efficiency of the drug. Multiple other studies have supported the significance of exosomes as both a therapeutic agent for treating diseases and a biological indicator following therapy, especially in cardiovascular disease [85].

## 9. Clinical Trials and Regulatory Considerations

### 9.1. Pre-Clinical Studies and Clinical Trials

Acellular therapies are comparatively new, and therefore, relatively few studies are available. There are few pre-clinical studies and initial-phase clinical trials that have been conducted over the last several years to investigate exosomes as therapeutic agents, which is indicative of the relatively new stage of scientific study in this emerging area [86]. Exosomes derived from MSCs offer great potential for clinical applications since they can be precisely delivered to the area of interest. Multiple ongoing pre-clinical studies in animal models have shown that MSC-derived exosomes have therapeutic effectiveness in treating and preventing diseases, including autoimmune diseases and neurological disorders, as well as their ability for cardiac, skin, and renal regeneration [87]. A randomized, placebo-controlled trial showed that exosomes obtained from adipose tissue-derived MSC (AT-MSC-exos) had a therapeutic effect on skin hyperpigmentation. A 0.2 g dose of AT-MSC-exos was administered to one group, and a 0.2 g placebo was given to the control group. The hyperpigmentation in the treatment group improved as a result of a transient decrease in melanin; nonetheless, relapses occurred subsequently [87]. A second published experiment demonstrated the efficacy of HUcMSM-exos in treating skin hyperpigmentation, with satisfactory results [88].

A clinical trial investigated the use of MSC-exosome therapy for patients with chronic kidney disease (CKD). Tubulointerstitial fibrosis, characterized by chronic renal function decline, is the primary characteristic of end-stage renal disease. Nassar et al. conducted a pioneering randomized controlled trial to investigate the effectiveness of exosomes produced from HucMSC-exos in reducing the development of chronic kidney disease (CKD) in stage 3 and 4 patients. The treatment group received a weekly dose of 100 mcg/kg body weight, whereas the control group was given a placebo. The first administration was delivered using the intravenous route, while the second dose was administered via the intra-arterial route. There was a considerable improvement in the estimated glomerular filtration rate and other indicators of renal function, such as blood creatinine and urea levels [89].

A summary of the registered clinical trials was collected from the www.clinicaltrials.gov website on 4 May 2024. The following searches were performed: “exosomes-based stem cell therapy” OR “EVs based stem cell therapy ” OR “exosomes in acellular therapy”. Table 2 shows some major clinical trials of exosome- based acellular treatments. Even though none of these investigations can be properly classified as regenerative medicine, these data are encouraging for potential future uses of exosomes. However, since several stem cell businesses are investing major efforts to create exosome-based therapies with stem cells, the lack of extensive published clinical studies in regenerative medicine does not represent all of the commercial and scientific interests.

### 9.2. Global Regulatory Requirements

The European Network on Microvesicles and Exosomes in Health and Disease and The International Society for Extracellular Vesicles have published recommendations to encourage its clinical usage because of the increasing therapeutic range of exosomal treatment [71]. The standard operating procedures (SOPs) that must be followed during the extraction, testing, processing, quality assurance, and manufacture of exosomes meant for use in clinical settings are described in depth in the rules. These regulations make it possible for exosomes to be utilized therapeutically within reasonable bounds. The development of new treatments or medicines is dependent upon process standardization strategies that give priority to technology validation. As of right now, no exosome product has received FDA approval for use by people in the United States. The FDA classifies exosomes as a 351 product, which means that additional research is required to confirm the safety and efficacy of products in treating diseases and that an IND may be required for clinical use. Therefore, any recently developed innovative exosome medicines for human use still present challenges [90].

## 10. Limitations of Exosome-Based Therapies

Exosomes provide a wide range of therapeutic possibilities because of their intrinsic characteristics of low immunogenicity and high biocompatibility. The advancement of novel exosome-based diagnostic tests and therapies is hampered by the limitations of currently available methods for isolating and characterizing exosomes. There is a large overlap in the density ranges of exosomes. The shape of exosomes is influenced by the techniques employed in the isolation process as well as the procedures employed for sample preparation and characterization. Various surface markers can be shown by exosomes produced from various cell types or under different conditions from the same cell type. For instance, CD63 is a recognized exosome marker, although it is absent from some exosome sub-populations [91]. Furthermore, exosomes have the potential to transport drugs to specific cells; however, there are currently limited techniques to control the packaging of cargo and release of vesicles in living cells. The manufacturing and storage costs are very high. In addition, there is a limited capacity to scale up exosome production for wider use [92]. There is currently no universally accepted procedure for isolating exosomes due to the diversity of available methods. Following the isolation of exosomes, the characterization process proceeds with quantifying the purity and yield, as well as the levels of proteins, lipids, and nucleic acids. If the sample fails to attain the required standards of purity and yield, additional analysis is necessary. The process of characterizing exosomes is also complex, as every isolation and characterization approach has its own advantages and disadvantages. The current methods prioritize the establishment of a universal set of exosomal marker proteins that are secreted by all cell types. It has also been shown that using various isolation methods to extract exosomes from the same type of cell leads to varying proteome profiles, which adds more complexity to the problem. It may be more advantageous to standardize marker proteins for all exosomes or select specific exosomal marker proteins for a particular cell type, irrespective of the isolation method [43].

The maintenance of exosomes remains difficult since these vesicles are most stable when stored for an extended period of time at temperatures close to −80 °C [93,94]. This requirement presents a secondary problem because low temperatures can reduce translational activity [95]. Freeze-drying of exosomes with vacuum by selectively removing water from a frozen specimen can overcome this problem. However, freeze drying can affect the exosome number, stability, and potency. In the freeze-drying cycle, the tendency of exosomes to aggregate can reduce their effectiveness. Stabilizers like starch or glucose can be added during the cryopreservation process to mitigate this effect [96]. Several studies have partially confirmed the impact of various storage conditions on exosomes, Sokolova et al. (2011) employed nanoparticle tracking analysis (NTA) to quantify the variations in exosome size under different temperature conditions. Their findings demonstrated that storing exosomes at 37 °C resulted in a greater reduction in size compared to storage at 4 °C. Nevertheless, this investigation did not provide any data on variations in particle concentration. Other studies have demonstrated that pH, storage temperature, and the number of freezing and thawing cycles have an impact only on the yield of the exosome, rather than on the quantitative changes. Thus, the established criteria for the preservation of exosomes remain unclear [97].

Exosomes generated from MSCs are small membrane vesicles that can modulate many biological processes involved in wound healing, such as cell proliferation, cell migration, and blood vessel development. Despite being a state-of-the-art ‘cell-free’ therapeutic alternative, MSC-derived exosomes treatment still faces several challenges that need to be addressed before it can be implemented in clinical settings. The therapeutic use of MSCs-derived exosomes is being hampered by the inconsistencies in the components of exosomes derived from different sources and the absence of established protocols for producing them on a large scale. Both of these limitations largely depend on quality assessments of the exosome sources. Currently, the use of MSC-exosomes in clinical settings is restricted due to the absence of a universally accepted standard of operation for cell culture conditions and procedures, exosome separation and storage, an optimal therapeutic dosage and administration schedule, and reliable potency tests to assess the effectiveness of exosome-based therapies [98,99].

Isolation, processing, and administration of exosomes under sterile conditions represent another potential issue when using exosomes as acellular treatments [100]. Exosomes can serve as biocarriers for retroviruses such as the human T-lymphotropic virus type 1 and the human immunodeficiency virus-1 during the biogenesis phase, allowing them to multiply throughout the body and avoid the immune system [101]. Viral material (such as retroviruses) can easily contaminate exosomes because of the minimal size difference between exosomes and viruses. Therefore, any pathogens should be closely monitored before clinical use to minimize the risks. Another important limitation of exosome use is the potential presence of oncogenic cargoes. For example, miRNAs are one of the major constituents of exosomes. Some miRNAs may have oncogenic potential, thereby promoting cell proliferation and leading to the development of tumors. Similarly, some proteins and enzymes contained in exosomes may stimulate pathways related to cancer. Since SC-exos are a relatively new topic, there is currently no standard protocol for isolating these vesicles, which can differ in quantity and quality and cause pleiotropic effects. This possibility puts exosomes at risk, in addition to raising concerns about safety and increasing the possibility of contamination. In addition, the long-term effects of exosomes in humans are still to be fully understood. It is essential to conduct further research to improve the isolation process while developing safe, standardized methods for purifying exosomes [86].

## 11. Conclusions and Future Perspectives

The use of exosomes in biomedical applications, both therapeutic and diagnostic, is made possible by their unique biological functions. These functions include facilitating intercellular communication, releasing exosomes with different types and effects depending on their origins and conditions, and presenting various markers on their membrane. By altering naturally occurring exosomes, the best nanocarriers can be synthesized to deliver therapeutic substances to target cells. However, there are limitations to exosomal-based acellular therapies, including exosome heterogeneity, uncertainty about the underlying mechanism of action, a lack of standardized isolation protocols, poorly understood and expensive characterization methods, and insufficient published data from clinical trials. Furthermore, there are currently limited techniques available to control the packaging of cargo and transport drugs to specific cells.

Further research into exosome biology is necessary to overcome the above limitations. To fully utilize the potential of exosomes, the development of new technologies must be integrated with basic research. Large-scale prospective research is needed to provide concrete evidence of exosome safety, viability, and pharmacodynamic properties. Moreover, delivery strategies beyond systemic dosing should be explored to target the therapeutic potential of exosomes. To optimize exosomes as drug carriers, it is essential to develop strategies that enhance their drug-loading capacity, specificity, safety, and immunological compatibility. Future pre-clinical research and clinical trials should involve a larger spectrum of disorders to better understand the therapeutic applications of exosome treatment. Upscaling exosome synthesis and multimodal exosome administration will accelerate the therapeutic application of stem cell-derived exosomes for many diseases.

## Figures and Tables

**Table 1:** Techniques for exosome isolation.

Sr. No.	Technique	Advantages	Disadvantages
1.	Differential centrifugation	Cost-effective, utilizes a single ultracentrifuge, needs minimum sample preparation.	Insufficient purification, contamination in resultant exosomes
2.	Polymer precipitation	Quick and easiy process, large volumes of samples can be processed	Low purity, difficulty in removing polymers.
3.	Exosomes isolation kits	Fast isolation procedure, no need for prior centrifugation or filtration, high yield and purity of resultant exosomes.	Isolation of large size exosomes, high cost, may not suitable for downstream applications.
4.	Sequential filtration	Streamlined and automated process, efficiently isolate exosomes from large volumes, preserved exosomal product	Time-consuming process, small exosomes can pass through the filters
5.	Immunomagnetic bead	High accuracy, no contamination, require low primary sample volume	Loss of recognition ability of specific antibodies, target exosomes may be lost
6.	Affinity chromatography	Isolate subpopulations of exosomes produced by specific cell types, visualization of individual exosomes.	Sample volume, non-specific binding, and purification challenge.

**Table 2:** Clinical trials of using exosomes for therapeutic applications.

NCT Number	Exosomes Source	Study Status	Conditions	Clinical Phase
NCT05669144	MSC-derived exosomes	Recruiting	Myocardial ischemia, myocardial infarction, myocardial stunning	Phase 1, Phase 2
NCT05402748	Human placenta MSC-derived exosomes	Recruiting	Fistula perianal	Phase 1, Phase 2
NCT05813379	MSC-derived exosomes	Recruiting	Anti-aging	Phase 1, Phase 2
NCT05413148	Stem cell exosomes	Recruiting	Retinitis pigmentosa	Phase 2, Phase 3
NCT05969717	Induced pluripotent stem cell-derived exosomes	Recruiting	Atopic dermatitis	Early phase1
NCT04276987	MSC-derived exosomes	Completed	Coronavirus	Phase 1
NCT05886205	IPSC-derived exosomes	Recruiting	Refractory focal epilepsy	Early phase 1
NCT06221787	Stem cell-derived exosomes	Recruiting	Melasma	NA
NCT05387278	Placental-derived exosomes	Recruiting	COVID-19 acute respiratory syndrome, Respiratory distress syndrome,	Phase 1
NCT05871463	MSC-derived exosomes	Recruiting	Liver fibrosis	Phase 2

## Abbreviations

CD: cluster differentiation; CKD: chronic kidney disease; DCs: dendritic cells; DLS: dynamic light scattering; EVs: extracellular vesicles; ESCRT: endosomal sorting complex required for transport; EGFR: epidermal growth factor receptor; FDA: food and drug administration; HucMSC-exos: human umbilical cord mesenchymal stem cell-derived exosomes; hESCs: human embryonic stem cells; IL: interleukin; IV: intravenous; ILVS: intraluminal vesicles; IM: intramuscular; miR: miRNA; MVBs: multivesicular bodies; MHC: major histocompatibility complex; MSCs: mesenchymal stem cells; NMDA: N-methyl-D-aspartate; NSCLC: non-small cell lung cancer; NTA: nanoparticle tracking analysis; PBS: phosphate-buffered saline; PEG: polyethylene glycol; RNP: ribonucleoprotein; SC: subcutaneous; SC-exos: stem cell-derived exosomes; TME: tumor microenvironment; TGF: transforming growth factor; VEGF: vascular endothelial growth factor.
